# Maternal characteristics and immunization status of children in North Central of Nigeria

**DOI:** 10.11604/pamj.2017.26.159.11530

**Published:** 2017-03-20

**Authors:** Olugbenga-Bello Adenike, Jimoh Adejumoke, Oke Olufunmi, Oladejo Ridwan

**Affiliations:** 1 Department of Community Medicine, Faculty of Clinical Sciences, College of Health Sciences, Ladoke Akintola University of Technology (LAUTECH), P.M.B. 4400, Osogbo, Osun State, Nigeria; 2 Federal Medical center, Keffi, Nasarawa State, Nigeria; 3 Department of Community Medicine, LAUTECH Teaching Hospital, P.M.B. 4008, Ogbomoso, Oyo State, Nigeria; 4 Institute of Education, department of Economics and Education, University of Ibadan, Oyo State, Nigeria

**Keywords:** Immunization status, children, childhood diseases, maternal characteristics, knowledge, communities, rural, urban, socio-demographic characteristics, education

## Abstract

**Introduction:**

Routine immunization coverage in Nigeria is one of the lowest national coverage rates in the world. The objective of this study was to compare the mother’ characteristics and the child’s Immunization status in some selected rural and urban communities in the North central part of Nigeria.

**Methods:**

A descriptive cross sectional study, using a multistage sampling technique to select 600 respondent women with an index child between 0-12 months.

**Results:**

Mean age of rural respondents was 31.40±7.21 years and 32.72+6.77 years among urban respondents, though there was no statistically significant difference in age between the 2 locations (p-0.762). One hundred and ninetyseven (65.7%) and 241(80.3%) of rural and urban respondents respectively were aware of immunization, the difference was statistically significant (p-0.016). knowledge in urban areas was better than among rural respondents. There was statistically significant association between respondents age, employment status, mothers' educational status and the child's immunization status (P<0.05), while variables like parity, age at marriage, marital status, No of children, household income and place of index were not statistically associated with immunization status as P>0.05. More than half 179(59.7%) of rural and 207(69.0%) of urban had good practice of immunization though the difference was not statistically significant (p-0.165)

**Conclusion:**

The immunization coverage in urban community was better than that of the rural community. The result of this study has clearly indicated that mothers in Nigeria have improved on taking their children for immunization in both rural and urban area compared to previous reports

## Introduction

Immunization is one of the most essential public health interventions and cost effective strategy to reduce childhood morbidity and mortality. It is estimated to prevent between 2 and 3 million deaths each year [1]. Despite this fact, vaccine-preventable diseases remain the most common cause of childhood mortality with an estimated three million deaths each year 2 [2]. Childhood immunization is the initiation of immunity through application of vaccine [3]. It is considered important for improving child survival [4]. This is because more than 10 million children in developing countries die every year because they do not access effective interventions such as immunization that could fight common and preventable childhood illnesses. Although, about three quarters of the world's child population is reached with the required vaccines, only half of the children in Sub-Saharan Africa get access to basic immunization [4]. Further, in poorer remote areas of developing countries, only one in twenty children have access to vaccination [5]. Immunization against vaccination preventable diseases (VPDs) through the expanded programme of Immunization (EPI) is one of the most economical public health interventions available6 that contributes extensively to achieving the Millennium Development Goal to reduce the mortality rate of children under five by two thirds between 1990 and 2015 [6, 7]. The EPI was established in 1974 against six vaccine preventable diseases. These are diphtheria, polio, tuberculosis, measles, pertussis and tetanus. In 2003, DPT3 global coverage was 78 percent with about 27 million children not covered. South Asia and sub-Sahara African countries accounted for 9.9 million and 9.6 million, respectively of the children that were not covered. In most of these countries poor functioning health service delivery system impedes the efforts to meet immunization targets [8]. Therefore, children living in remote location and border areas are difficult to be reached. Other areas not reached were displaced populations. Also, some people lack access to vaccination due to social barriers, lack of information or inspiration to get vaccinated [9].

Nigeria started its EPI in 1979 (NPI, 2001). Reports have shown that the coverage has been fluctuating especially after the global universal childhood immunization efforts ended in 1990. This could have resulted from low political will and social support, inadequate funding and poor community involvement and participation. In view of the important need to improve the efficiency of immunization which was declining so fast and also to meet the universal challenges of immunization, the EPI programme was re-visited and re-named National Programme on Immunization (NPI) in 1995, which probably accounted for observed increase in coverage after the period (17% in 1999, 13% in 2003 and 23% in 2008) (NPI, 2005; 2008). In 2000, WHO/UNICEF estimated that DPT3 coverage for children between under 1 year 10-12 months was 23%, polio third dose was 26% and measles containing vaccines (MCV) was 33%. By 2009, the estimates were respectively 42% (DPT3), 54% (Pol3) and 41% (MCV). Routine immunization coverage in Nigeria is one of the lowest national coverage rates in the world with 38% for January-December 2005 and 50% January- May 2006 (NPI, 2005; 2006). In recent time, scholars working on child health in Nigeria have started documenting various factors militating against child survival and ethnic differentials in under-5 mortality in the country [10].

Also, the first round results of a 2006 national immunization coverage survey reported only 18% of children fully immunized aged 10-12 months at survey time [11]. According to UNICEF, the WHO, and National Programme on Immunization (NPI) guiding principle, in Nigeria a child receives a Bacille Calmette-Guerin (BCG) vaccination for tuberculosis, four doses of oral polio, three doses of DPT (diphtheria, pertussis, and tetanus), and one dose of measles vaccine by age 12 months [12]. But because children below age two are vulnerable to illnesses due to weak immune system, they are regularly immunized with booster doses especially of polio and measles during the national or sub national immunization days. In Nigeria, vaccination is given on routine and outreach bases. According to the Expanded Programme on Immunization, a routine vaccination schedule for children in Nigeria are given starting from birth, and are being completed before one year of life by all children (WHO and UNICEF, 2008). BCG and OPV0 are administered at birth, while three doses of OPV and pentavalent vaccines (which protect against diphtheria, pertussis, tetanus, hepatitis B and Haemophilus influenzae type B diseases) are given at interval of four-week duration; at 6, 10 and 14 weeks, and measles vaccine is given at the age of nine months) [13].

Less than half of children have received each of the recommended vaccinations, with the exclusion of polio 1 (67 per cent) and polio 2 (52 per cent) (NPC, 2003). And more than three times as many urban children as rural children are fully vaccinated (25 per cent and 7 percent, respectively) (NPC, 2003). It is therefore important to study the factors associated with full child immunization in Nigeria with a view to improve the quality of life of these children. Nigeria's immunization coverage rates are among the worst in the world [12]. According to the 2003 National immunization schedule, the percentage of fully immunized children to be targeted was less than 1% in Jigawa, 1.5% in Yobe, 1.6% in Zamfara and 8.3% in Katsina. It was also revealed that only 23% of Nigeria children 12-23 months received all recommended vaccines as at 2008 that is one dose of BCG and measles and three doses each of DPT and polio (NPC 2008). The same survey showed that 38 percent of children in Nigeria had not received any vaccinations. As a result, thousands of children are dying as victims of vaccine preventable diseases. Several studies have examined issues on child immunization in Nigeria including patterns of uptake as well as determinants [14]. However, these studies have been limited to a selection of vaccines and often at local geographic areas. Identifying the determinants of full childhood vaccination in a representative sample of Nigeria population is lacking. Identifying the factors that determine full child immunization in a representative sample of the country will enable the government to provide programmes and service that focused on the environment through well-articulated policies, projects and programmes and good standard of practice. The study was aimed to compare the Immunization status and dropout rate among children between 0-12 months in some selected rural and urban communities in the North central part of Nigeria.

## Methods

The town of keffi, is located in Nasarawa state, in the central region of Nigeria, lying south-west of the plateau, about fifty miles north of the Benue River and forty miles west of the Trunk 'A' road from Jos through Akwanga, Lafia, Makurdi, Oturkpo down to the Eastern states. It serves as the gate to the North western states. The population of keffi as at 2006 was estimated be 92, 664. The climate is characterized by a tropical sub-humid climate with two district seasons. The economic activities of the inhabitants of keffi are trade, commerce and essentially agro-allied industry. Seventy percent of the inhabitants are farmers in food crops such as grains, yam tubers, Fruits and vegetables and cash crop such as cocoyam, rice and corn, the remaining 30% are involved in petty, trading, cattle rearing, and civil services. There are nine (9) public Primary Schools, two (2) government secondary schools, and six (6) private nursery and primary school in the community. Keffi had two (2) Primary health centers, one (1) comprehensive health centre, and one (1) tertiary health centre, with four (4) Private Hospitals and many traditional birth attendants posts Islam is the dominant religion, while Christianity and Traditional religions are practiced according to the conviction of individuals. It has a predominantly Fulani speaking population, however many other Nigerian tribes such as Hausa, Gwandara, Mada, Yeskwa, Gwari Yoruba and Igbo settled in large numbers within the community.

### Sample size determination

The sample size was calculated using the formula for comparing two groups:

$$\frac{{\text{N=2(Zα+Zβ)}}^{2}{\text{P}}_{0}{\text{(1-P}}_{0})}{{\text{d}}^{2}}$$

Where; N= Minimum sample size

Zα= critical ratio at significance level of 5%, which is 1.96

Zβ= statistical power at 90%, which is 1.28

P_0_= means of the 2 prevalence's of the two comparison groups i.e. (P1+P2)/2

d= differences between P1 and P2 Based on previous documented studies done in Nigeria, the proportion of fully immunized children in rural and urban communities respectively. Therefore, the prevalence in the two groups (P1 and P2) was taken to be 42.95% [15] urban area and 29.5% [16] rural.

P_0_ = 0.753+ 0.849/2 = 0.801

d= differences between P1 and P2: (0.849-0.753) = 0.096

$$\frac{{\text{N=2(Zα+Zβ)}}^{2}{\text{P}}_{0}{\text{(1-P}}_{0})}{{\text{d}}^{2}}$$

$$\text{N}=\frac{2{\left(1.96+1.28\right)}^{2}0.36225(1-0.36225)}{0.1345^{2}}$$

= 268

= 268 respondents

The calculated minimum sample size is 268 respondents each for the selected rural and urban communities. After taking care of an anticipated non-response rate of 10%, the minimum sample size increased to 270 respondents, but 300 respondents each from the selected urban and rural communities will be interviewed to enhance representativeness (i.e. total sample size of 600) A multi stage sampling technique was used to select 300 consenting Women of reproductive age group each from the rural and urban communities, having an index child within the age 0-12 months in Keffi Local government area. The main instrument used for this study was a pre-tested, semi-structured, interviewer administered questionnaire. The questionnaire was divided into 4 sections aimed at covering the scope of the study, section I: socio-demographic status of the respondent; section II: knowledge of respondent about immunization; section III: attitude of the respondent to immunization and section IV: immunization practices by the respondents. The questionnaires were pre-tested among mothers of Akwanga a town outside keffi. This was to ensure validity and reliability of the instrument. The pre-tested questionnaires were analyzed and necessary modifications were put into effect. After approval to carry out the study by the local government authority and the traditional ruler of the communities, a verbal informed consent was sought from the respondents after detail explanation of the research was made. Only respondents who gave their consents as well as met the inclusion criteria were interviewed.

The data collected were manually sorted; mothers' knowledge on the age a child should start immunization was re-categorized based on the responses from the respondents. at birth was categories as correct response while 24 hours after birth, 21 days, 28 days, 41 days and 0-1years was categories as incorrect, and those who did not respond to the question was categories as 'don't know'. However, the mothers' knowledge on the age a child should complete the immunization schedule was also re-categories based on the responses generated by the respondents, those who said 9months was categorized as correct response, those who said 3, 6, and 12 months was categorized as incorrect while those who did not respond to the question was categorized as 'don't know'. The mothers' knowledge on the diseases against which NPI is scheduled was scored on the maximum score of responses 9 point, those who scored <5 was categorized as poor knowledge and those that scored <5 was categorized as good knowledge. Moreover, the mothers' knowledge on the names of vaccines was scored on the maximum score of responses 7points, those who scored <4 was categorized as poor knowledge while those who scored ≥4 was scored as good knowledge. The knowledge of respondents on immunization schedule currently practiced in Nigeria was scored on scale of 6 points, those that scored ≤3 was categorized as poor knowledge and those that scored >3 were categorized as good knowledge. Generally, mothers' knowledge on immunization was scored on the scale of 0-1 based on the range of responses obtained from respondents on the following questions. Are you aware of immunization, are vaccine harmful, what do you know about immunization, what age should a child be immunized, can a child with fever be immunized, means of awareness about immuzation and vaccination. Those who give positive response were scored 1 those who gives negative response were scored 0. Aggregate for score each respondent was found to be maximum score of 18. This was done based on the accuracy of the range of responses given by the respondents. Score of ≤9 was rated as poor knowledge and > 9 was rated good knowledge. The practice of immunization was scored using the respondents' responses to 10 variables. Range of responses is 'YES' and 'NO'. Each response was assigned a score from 1-0 in the order of response, responses of respondents were categories as poor practice and good practice of immunization, aggregate score for each respondent was 10 points. Those that scored 0 -5 were scored poor practice, while those that scored >5 were scored good practice.

## Results

Table 1 showed the socio-demographic characteristics of the respondents Mean age of rural respondents was 31.40±7.21 years and 32.72+6.77 years among urban respondents, though there was no statistically significant difference in age between the 2 locations (p-0.762). Age group 26-30 years constituted the highest age proportion in both locations with 91(30.3%) in rural and 97(32.3%) among the rural. While there is statistically significant difference in parity between locations (p 0.028), mean parity was 3.12±1.55 among rural and 2.51+1.34 among urban respondents. There was no statistically significant difference in marital status, No of children, age of child, Employment status, maternal education level between rural and urban respondents(p>0.05). However, there was statistically significant differences in Place of birth for index child, sex of the child and Place of birth for index child between rural and urban locations (p<0.05). Table 2 showed the respondents family bio-data. There is no statistical difference on household income and family settings between the two groups (p-0.394). On religion status, there was significant difference between the two groups (p-0.002) and majority 181(60.3%) were Muslim in rural while 184(61.3%) were Christian in urban area. Concerning the father ethnicity group and nationality, there was no significant different (p 0.385, 0.537) and all the responses were of the same proportions. Moreover, majority 171(57.0%) of the child in rural were male while 185(61.7%) in urban were female and there was statistically difference (p-0.008). the child age and their birth order among both rural and urban dwellers was not significant as (p-0.778, 0.509).

**Table 1:** Socio-demographic characteristics of mothers (N=600)

Bio data	RURAL (n=300)	URBAN (n=300)	X2	df	P-value
	Frequency (%)	Frequency (%)			
**Age(years)**	31.40±7.21	32.72+6.77	0.092	5	0.762
< 20	9(3.0)	6(2.0)			
21-25	67 (22.3)	44(14.7)			
26-30	91 (30.3)	97(32.3)			
31-35	33 (11.0)	45(15.0)			
36-40	70(23.3)	72(24.0)			
>40	30(10.0)	36(12.0)			
Parity	3.12±1.55	2.51+1.34	4.83954	2	^+^0.028
1-2	136(45.3)	182(60.7)			
3-4	111(37.0)	90(30.0)			
>4	53(17.7)	28(9.3)			
**Age at marriage**	22.46±6.51	23.35+7.42	3.409	2	^+^0.045
<18	85(28.3)	52(17.3)			
19-25	135(45.0)	176(58.7)			
>25	80(26.7)	72(24.0)			
**Marital status**			1.797	2	0.180
Married	289(96.3)	269(89.7)			
Separated	6(2.0)	8(2.7)			
Widowed	5(1.7)	23(7.7)			
**No of children**			0.563	2	0.453
1-3	230(76.7)	225(75.0)			
4-6	11(3.7)	64(21.3)			
>6	59(19.7)	11(3.6)			
**Place of birth for index child**			5.835	2	^+^0.016
Health facility	155(51.7)	205(68.3)			
Home	67(22.3)	43(14.3)			
TBA	78(26.0)	52(17.3)			
**Employment status**			0.012	3	0.914
Full house wife	142(47.3)	131(43.7)			
Trader	61(20.3)	103(34.3)			
Farming	46(15.3)	21(7.0)			
Working class	51 (17.0)	45(15.0)			
**Maternal education level**			0.003		0.951
No formal education	73 (24.3)	66(22.0)			
Primary	95 (31.7)	90(30.0)			
SecondaryTertiary	67(22.3) 65 (21.7)	93(31.0) 51(17.0)			

+ Statistically significant

**Table 2:** Child’s bio-data (n=600)

Variables	RURAL (n=300)	URBAN (n=300)	X2	df	P-value
	Frequency (%)	Frequency (%)			
**Household income**			0.728	3	0.394
>10000	76(25.3)	47(15.7)			
>50000	167(55.7)	191(63.7)			
<100000	26(8.7)	43(14.3)			
>100000	31(10.3)	19(6.3)			
**Family setting**			0.011	1	0.916
Monogamous	217(72.3)	215(71.7)			
Polygamous	83(27.7)	85(28.3)			
**Religion**			9.4733		^+^0.002
Christianity	114(38.0)	184(61.3)			
Islam	181(60.3)	109(36.3)			
Traditional	5(1.7)	7(2.4)			
**Fathers ethnicity**			0.756	3	0.385
Yoruba	42(14.0)	73(24.3)			
Hausa	107(35.7)	171(57.0)			
Igbo	48(16.0)	38(12.7)			
Others	103(34.3)	18(6.0)			
**Nationality**			0.381	1	0.537
Nigerian	298(99.3)	295(98.3)			
Non-Nigerian	2(0.7)	5(1.7)			
**Sex of the child**			7.054		^+^0.008
Male	171(57.0)	115(38.3)			
Female	129(43.0)	185(61.7)			
**Age of Child**			0.079	2	0.778
<6months	171(57.0)	176(58.7)			
0-12months	66(22.0)	95(31.7)			
6-12 months	63(21.0)	29(9.7)			
**Birth order**			0.435	2	0.509
1^st^	69(23.0)	35(11.7)			
2^nd^	87(29.0)	147(49.0)			
3^rd^& above	144(48.0)	118(39.3)			

+ Statistically significant

**Figure 1:**
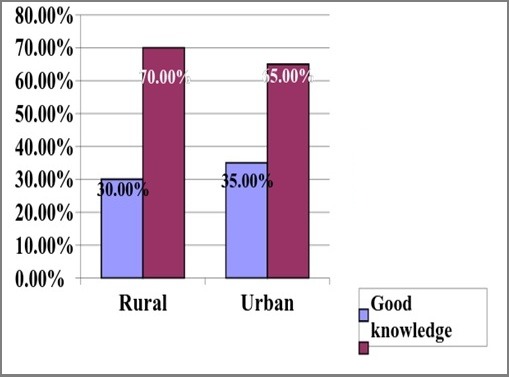
Maternal Knowledge score on Disease for scheduled NPI (X2 =0.563, p-value=0.453, Remarks= Not significant)

**Figure 2:**
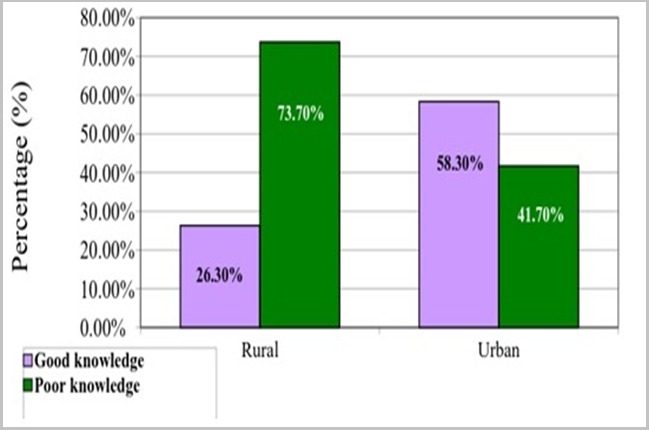
Maternal knowledge score on names of vaccines

Table 3 shows the maternal knowledge on immunization. One hundred and ninety-seven (65.7%) and 241(80.3%) of rural and urban respondents respectively were aware of immunization, though the difference was statistically significant(p-0.016). The various media of awareness was adopted by the 2 groups of respondents while several reasons were given for taking immunization. Seventeen (5.7%) and 24(8.0%) said vaccines are harmful. There was no statistically difference in the knowledge of when a child should start and completes immunization, among rural and urban respondents (p>0.05). However, there was a statistically significant difference in the knowledge of the diseases being vaccinated against among rural and urban respondents (p-0.009) and likewise the names of the vaccines (p-0.004). For these 2 variables, knowledge in urban areas were better than among rural respondents. However, there were no statistically significant differences in the knowledge of symptoms of immunization among rural and urban respondents (0.097). Figure 1 shows the respondents knowledge of immunization schedule in Nigeria. Knowledge score on Disease for scheduled NPI was good among 90(30.0%) of rural and 105(35.0%) of urban respondents and this shows that there was no difference between the two groups (p-0.453). Figure 2 shows the respondents knowledge on names of immunization in Nigeria. it was showed that Knowledge of mother on names of vaccines was good among 79(26.3%) of rural and 175(58.3%) of urban respondents. there was statistical difference between the two groups (p-0.024).

**Table 3:** Mothers’ knowledge on immunization (N=600)

Variables	RURAL(n=300)	URBAN(n=300)	X2	df	P-value
	Frequency (%)	Frequency (%)			
**Awareness of immunization**			5.795	1	^+^0.016
Yes	197(65.7)	241(80.3)			
No	103(34.3)	59(19.7)			
**What do you know about it**			13.231	4	^+^<0.001
a drug given to children	38(12.7)	51(17.0)			
A vaccine to prevent diseases	110(36.6)	87(29.0)			
Improve children immune	72(24.0)	55(21.3)			
It’s an injection	17(5.7)	89(29.7)			
Don’t know	63(21.0)	18(6.0)			
**Reasons for immunization**			1.036	3	0.311
For good health	110(36.6)	119(39.7)			
For child development	65(21.7)	77(25.7)			
Prevent communicable diseases	42(14.0)	57(19.0)			
Don’t know	83(27.7)	47(15.6)			
**Are vaccines harmful**			2.698	2	0.101
Yes	17(5.7)	24(8.0)			
No	179(59.7)	232(77.3)			
Don’t know	104(34.7)	44(14.7)			
**What age should a child be immunized**			1.231	2	0.268
Correct	189(63.0)	223(74.3)			
Incorrect	17(5.7)	23(7.6)			
Don’t know	97(32.3)	55(18.3)			
**Age to complete immunization**			0.011	2	0.917
Correct	103(34.3)	185(61.7)			
Incorrect	81(27)	49(16.3)			
Don’t know	116(38.7)	66(22.0)			
**Can children with fever be immunize**			12.305	2	^+^<0.001
Yes	83(27.7)	189(63.0)			
No	96(32.0)	36(12.0)			
Not sure	121(40.3)	75(25.0)			
**^++^Symptoms of immunization**			2.741	5	0.097
Fever	157(52.3)	187(62.3)			
Body Pain	177(59.0)	165(55.0)			
Rash	85(28.3)	36(12.0)			
Body weakness	75(25.0)	40(13.3)			
Body swelling	96(32.0)	48(16.0)			
Diarrhea	57(19.0)	79(26.3)			
**^++^Means of awareness**			0.007	7	0.933
Radio	119(39.7)	186(62.0)			
Television	88(29.3)	125(41.7)			
News paper	33(11.0)	76(25.3)			
Friends	27(9.0)	19(6.3)			
School	36(12.0)	43(14.3)			
Health workers	160(53.3)	182(60.7)			
Community leaders	58(19.3)	23(7.7)			
Symposium/lecture	19(6.3)	25(8.3)			

++ Multiple responses ;

+ statistically significant

Table 4 showed practice of immunization among respondents. One hundred and eighty-two (60.7%) of rural and 246(82.0%) on urban respondents took vaccinations for their previous children and the difference was statistically significant (p-0.002) with better practice among the urban respondents. One hundred and sixty-eight (56.0%) of rural and 247(82.3%) of urban respondents completed their child immunization, and the difference was statistically significant (p-0.001) with better practice among the urban respondents. One hundred and seventy-two (57.3%) of rural and 213(71.0%) of urban respondents completed their child immunization according to the Nigerian schedule and the difference was statistically significant (p-0.004) with better practice among the urban respondents. Several reasons were given by respondents in both locations for not completing immunization including nearness to the health center, and side effects of vaccines which were reported to the doctor/HCW by a significant proportion of the respondents in both locations. Table 5 revealed the immunization details of the respondents. More than a third, 223 (74.1%) of the urban respondents had immunization card compared to 168(56.0%) of the rural, the difference was statistically significant (p-0.005). About 34(24.6%) were never immunized among rural while 33(14.8%) were never immunized among urban. Table 6 shows the association between socio-demographic characteristics and respondents' child immunization status in rural area. It shows that there were statistical significance associations between respondents age, employment status in association with immunization status (P<0.05) while variables like parity, age at marriage, marital status, No of children, maternal education level, household income and place of index were not statistically associated with immunization status as (P>0.05). Table 7 shows the association between socio-demographic characteristics and respondents' child immunization status in urban area. It shows that there were statistical significance associations between respondents age, employment status, maternal education level and household income in association with immunization status as (P<0.05) while variables like parity, age at marriage, marital status, No of children, and place of index were not statistically associated with immunization status as (P>0.05). The proportion of mothers in the urban communities who had good mean knowledge scores about immunization were more than those in the rural communities, (187(62.3%) in the urban, and 132(44.0%) in the rural). This difference was statistically significant (p-0.009). However, 207(69.0%) of urban and 179 (59.7%) of rural had good mean practice score of immunization and the difference was not statistically significant (p-0.165).

**Table 4:** Practice of immunization among mothers

Variables	RURAL(n=300)	URBAN(n=300)	X2	df	P-value
	Frequency (%)	Frequency (%)			
**When you started vaccination for child**			0.782	2	0.376
At birth	124(41.3)	161(53.7)			
others	11(5.3)	9(3.0)			
Don’t know	165(55.0)	130(43.3)			
**Were previous children immunized**			10.034	1	^+^0.002
Yes	182(60.7)	246(82.0)			
No	118(39.3)	60(18.0)			
**Was their immunization complete**			17.787	1	^+^<0.001
Yes	168(56.0)	247(82.3)			
No	132(44.0)	53(17.7)			
**If no, why**	n=132	n=53	3.155	7	0.055
It’s against my religion	25(18.9)	11(20.7)			
Attitude of the health workers	13(9.8)	4(7.5)			
Cultural belief	31(23.5)	7(13.2)			
Afraid	12(9.1)	2(3.8)			
Lack of interest in it	18(13.6)	5(9.5)			
Far distance of health centre	11(8.3)	21(39.6)			
Absents of health workers	3(2.3)	0(0.0)			
Time constraint	19(14.5)	3(5.7)			
**Distance of house and primary health centre**			0.053	3	0.818
Very near	16(5.3)	18(6.0)			
Near	94(31.3)	162(54.0)			
Far	108(36.0)	74(24.7)			
Very far	82(27.3)	46(15.3)			
**What is the next immunization**			2.807	5	0.094
Measles	57(19.0)	39(13.0)			
Yellow fever	12(4.0)	25(8.3)			
Vitamin A	22(7.3)	31(10.3)			
Polio	6(2.0)	27(9.0)			
Pental	0(0.0)	2(0.7)			
None	203(67.7)	176(58.7)			
**completion of child immunization according to schedule**			4.177	1	^+^0.041
Yes	172(57.3)	213(71.0)			
No	128(42.7)	87(29.0)			
**If yes, was any side effect noticed**	n=172	n=213	10.201	1	^+^0.004
Yes	68(39.5)	31(14.6)			
No	102(59.3)	182(85.4)			
**If yes, which were seen	n=172	n=213	0.054	5	0.817
Pain	43(25.0)	22(10.3)			
Fever	28(16.3)	19(8.9)			
Swelling					
Rash	23(13.4) 7(4.1)	13(6.1) 6(2.8)			
Others(vomiting)	5(2.9)	1(0.3)			
**Did you inform doctor/healthcare workers**	n=48	n=31	0.605	1	0.436
Yes	20(41.7)	17(54.8)			
No	28(58.3)	14(45.2)			
**If yes, what was done**	n=20	n=17	0.775	1	0.379
Provide medicine for illness	14(70.0)	8(47.1)			
Provide treatment for illness	6(30.0)	9(52.9)			

++ multiple responses;

+ Statistically significant

**Table 5:** Child’s Immunization bio-data in rural and urban area compared

Variables	Rural	Urban	X^2^	Df	P-value
**Does the child have an immunization card**					
Yes	168(56.0)	223(74.1)	7.732	1	^+^0.005
No	132(44.0)	77(25.9)			
**Does the child have a BCG scare**			3.199	1	0.074
Yes	163(54.3)	130(44.4)			
No	137(45.7)	170(56.8)			
**BCG OPVo HBV**			0.663	1	0.416
Yes	169(56.3)	203(67.9)			
No	131(43.7)	97(32.4)			
**PENTAL 1OPV1**			0.009	1	0.926
Yes	180(60.0)	189(63.0)			
No	120(40.0)	111(37.0)			
**PENTAL 2 OPV2**			0.010	1	0.921
Yes	169(56.3)	193(64.2)			
No	131(43.7)	107(35.8)			
**PENTAL 3 OPV3**			0.537	1	0.464
Yes	153(51.1)	170(56.6)			
No	147(49.7)	130(43.4)			
**Measles**			1.234	1	0.268
Yes	132(44.0)	147(49.0)			
No	168(56.0)	153(51.0)			
**Yellow fever**			0.039	1	0.843
Yes	121(40.3)	84(28.0)			
No	179(59.7)	216(72.0)			
**Immunization status**	n=138	n=223	0.749	2	1.528
Never immunized	34(24.6)	33(14.8)			
Fully immunized	63(45.7)	149(66.8)			
Partially immunized	41(29.7)	40(17.9)			

+ Statistically significant

**Table 6:** Association between mothers’ socio-demographic characteristics and respondents’ child immunization status in urban area

Bio data	Fully Immunized	Partially immunized	Never immunized	X2	Df	P-value
**Age(years)**				12.43	15	^+^0.004
< 20	5(71.4)	2(28.6)	0(0.0)			
21-25	25(55.6)	14(31.1)	6(13.3)			
26-30	29(63.0)	7(15.2)	10(21.7)			
31-35	21(72.4)	8(27.6)	0(0.0)			
36-40	51(69.9)	6(8.2)	16(22.2)			
>40	18(78.3)	3(13.0)	2(8.7)			
**Parity**				3.068	6	0.079
1-2	71(62.8)	20(17.7)	22(19.5)			
3-4	51(72.9)	10(14.3)	9(12.9)			
>4	27(67.5)	10(25.0)	3(7.5)			
**Age at marriage**				2.218	6	0.136
<18	34(55.7)	18(29.5)	9(14.8)			
19-25	61(70.1)	13(19.9)	13(19.9)			
>25	54(72.0)	9(12.0)	12(16.0)			
**Marital status**				0.764	6	1.356
Married	143(65.9)	40(18.4)	34(15.7)			
Separated	3(100.0)	0(0.0)	0(0.0)			
Widowed	3(100.0)	0(0.0)	0(0.0)			
No of children				0.464	9	0.496
1-3	51(69.9)	9(12.3)	13(17.8)			
4-6	58(65.9)	18(20.5)	12(13.6)			
>6	50(69.4)	13(18.1)	9(12.5)			
**Employment status**				8.208	9	^+^0.004
Full house wife	48(53.3)	19(21.1)	23(25.6)			
Trader	47(78.3)	6(10.0)	7(11.7)			
Farming	20(74.1)	7(25.9)	0(0.0)			
Working class	34(73.9)	8(17.4)	4(8.7)			
**Maternal educationlevel**				0.036	9	0.851
No formal education	15(48.4)	8(19.5)	8(25.8)			
Primary	38(56.7)	16(23.9)	13(19.4)			
Secondary	52(74.8)	9(12.9)	9(12.9)			
Tertiary	44(80.0)	7(12.7)	4(7.3)
**Household income** >10000	42(77.8)	9(16.7)	3(5.6)	1.136	9	0.288
>50000	64(59.8)	20(18.7)	23(21.5)			
<100000	19(70.4)	4(14.8)	4(14.8)			
>100000	24(68.6)	7(20.0)	4(11.4)			
**Place of index**				0.834	6	0.361
Health facility	103(72.0)	20(14.0)	20(14.0)			
Home	32(58.2)	12(21.8)	11(20.0)			
TBA	14(56.0)	8(32.0)	3(12.0)			

+ Statistically significant

**Table 7:** Association between mothers’ socio-demographic characteristics and child’s immunization status in rural area

Bio data	Fully Immunized	Partially immunized	Never immunized	X2	Df	P-value
**Age(years)**				35.601	15	^+^0.004
< 20	3(75.0)	1(25.0)	0(0.0)			
21-25	8(32.0)	12(48.0)	5(20.0)			
26-30	19(59.3)	3(9.4)	10(31.3)			
31-35	7(36.8)	8(42.1)	4(21.1)			
36-40	13(38.2)	11(2.9)	10(29.4)			
>40	13(54.2)	6(25.0)	5(20.8)			
**Parity**				1.053	6	0.307
1-2	32(38.6)	24(28.9)	27(32.5)			
3-4	23(52.3)	16(36.4)	5(11.4)			
>4	8(72.7)	1(9.1)	2(18.2)			
**Age at marriage(years)**				1.274	6	0.259
<18	19(45.2)	13(30.9)	10(23.8)			
19-25	25(43.9)	20(35.1)	12(21.1)			
>25	19(48.7)	8(20.5)	12(30.8)			
**Marital status**				2.988	6	0.084
Married	58(46.0)	35(27.8)	33(26.2)			
Separated	1(100.0)	0(0.0)	0(0.0)			
Widowed	4(36.4)	6(54.5)	1(9.1)			
**No of children**				0.003	9	0.986
1-3	41(47.7)	23(26.7)	22(25.6)			
4-6	18(41.9)	18(41.9)	7(16.3)			
>6	4(44.4)	0(0.0)	5(55.6)			
**Employment status**				9.698	9	^+^0.002
Full house wife	25(41.7)	13(21.7)	22(36.7)			
Trader	14(37.8)	17(45.9)	6(16.2)			
Farming	9(69.2)	4(30.8)	0(0.0)			
Working class	15(53.6)	7(25.0)	6(21.1)			
**Maternal education level**				7.644	9	^+^0.006
No formal education	8(40.0)	2(10.0)	10(50.0)			
Primary	19(44.2)	14(32.6)	10(23.3)			
Secondary Tertiary	18(43.9) 18(52.9)	16(39.0) 9(26.5)	7(17.1) 7(20.6)			
**Household income**				5.799	9	^+^0.016
>10000	20(69.0)	8(27.6)	1(3.4)			
>50000	23(31.5)	29(39.7)	21(28.8)			
<100000	11(52.4)	4(19.0)	6(28.6)			
>100000	9(60.0)	0(0.0)	6(40.0)			
**Place of index**				0.276	6	0.599
Health facility	42(43.8)	31(32.3)	23(23.9)			
Home	13(52.0)	3(12.0)	9(36.0)			
TBA	8(47.1)	7(41.2)	2(11.7)			

+ Statistically significant

## Discussion

### Statement of principal finding

The majority of the respondents, both at the rural and urban areas were within the age range 26-30 years. The mean age was 31.40±7.21 years in the rural area and 32.72+6.77 years in the urban area. The study observed a higher parity among the rural women, this observation corroborates the findings of Adebowale et-al, that documented better access to family planning programs among the urban women than their rural counterpart, which was associated with reduced fertility in urban areas [17]. There were more educated women in the urban communities compared to the rural communities, and mothers' education was found to be significantly associated with child's immunization status. This finding is consistent with that observed by Tadesse et al, 2009 [18] and Breiman et al. 2004 and other researchers [1, 19, 20] reported that mother's education was a significant predictor of completeness of immunization because highly educated mother will be more aware of the importance of immunization [21]. In Papua New Guinea, It has been observed that higher mother's education was associated with the knowledge of when to start immunizations, the frequency of visits and the diseases prevented by each vaccine [22], which was also supported by this study wherein, Higher education attainment was associated with good knowledge about immunization, which also was associated with the child been fully immunized. [23] High level of awareness about immunization was observed in this study among both the rural and urban communities. This is comparable to findings in a rural community based study in Edo State, Nigeria (99.9%) [24], 99.8% in Kinshasa, DRC [25], 96% reported in Ethiopia [26], and 93.8% reported by a facility based study in urban Lagos [27]. The health care givers should be commended as they were the major source of information to the study population.

Mother´s occupation is another factor that influence vaccination uptake. Majority of the respondents in both rural and the urban area were full housewives, however, this study observed and supported the findings by Antai, 2009 that being employed was significantly associated with higher likelihood of the child being fully immunized [28]. There was statistically significant difference in Place of birth for index child between rural and urban locations (p<0.05). It was observed that more of the children from the rural setting were delivered at home and TBA facilities than in health facilities. This finding supports the findings by a study done in Bangladesh, which shows that home delivery by TBAs remain the first preference for pregnant women in rural areas [29]. Poverty is the most frequently cited reason for preferring home delivery with a TBA. The patronage of TBAs at the rural communities is a major reason for the lower proportion of fully immunized children observed compared to the urban communities, as the place of delivery was significantly associated with immunization status in this study which was also observed in the study conducted in Niger Delta area of Nigeria by Oyo-ita et al, 2012 [30] and others [31, 32]. A child that was delivered in a health facility would have better access to immunization than a child born at home or TBA centre. At birth a child is given OPVo, BCG and HBV1 and this allows the mother to be informed about other components of routine immunization and their importance. Travel time to health facility has been observed to be a barrier to delivery of infant vaccines in remotes areas. This statement was supported by this study and several others [16, 23, 33]. Distance to primary health care facilities was significantly associated with immunization status of the children. This may be related to socioeconomic factors and cost of transportation for each immunization session especially if health care facilities are not in close proximity.

This study shows that majority of the respondents were from families where the monthly income is greater than N50, 000, indicating a middle-class standard of living, (Renaissance Capital, 2011). Several studies have found a relationship between wealth status and vaccination status [14, 34]. Children from wealthy households may be more likely to have their vaccination status checked and to receive missing doses of vaccines when attending a health care facility than children from poor households. Also, it could be because children who are from poor homes find it difficult to be reached by the health workers and also poor parents may encounter barriers to reach health facility compared to rich children. A large percentage of the respondents in both rural and urban area said vaccines are not harmful, they had good knowledge of when a child should start and completes immunization, they also had good knowledge of disease being vaccinated against; they knew the names of the vaccines, with no statistical difference. The result of this study was similar to other findings in an Italian study [35] in which 57.8% of parents had adequate knowledge-attitude-practice (KAP), and is supported by a study in India [36] that found parental knowledge regarding vaccination adequate. Also, this study observed that the age of the mother is significantly associated with the child's immunization status, (p>0.004) in both rural and urban area respectively. This could be because older mothers know the effect and the importance of immunization on children than young women. This finding is the same with the study conducted in Sudan by Ibnouf et al, 2007 [37] and also in Nigeria by Babalola 2009 [14].

More children were fully immunized in the urban communities, compared to the rural. This is probably partially due to the general distribution of health care facilities in the country which tend to favor large number of people in the urban area of the country. It could also be attributed to the lack of awareness of the importance of vaccination between mothers in rural areas in comparison to those in urban areas. The UN General Assembly special session on children set targets of full immunization of children under one year at 90 percent nationally. Attainment of at least 80 percentage coverage in measles can promote hard immunity for the children below the age of five. Though Harper (1962) postulates that complete immunization of every susceptible individual is not practicable in a free community like Nigeria, however Lower immunization dropout rates may be achieved by making vaccines readily available and fostering community mobilization and participation Assessment of mothers' knowledge on importance of vaccination and vaccine preventable diseases was moderately high in both rural and urban areas as observed by this study, this could account for the proportion of children who were fully immunized in the study. Other studies have also shown that a mother's knowledge of the importance of vaccination has a strong relationship with complete immunization status of children [18, 38]. Other researchers have established that a mother's knowledge of the immunization schedule has a strong association with complete immunization status of children [26, 39]. However, the mothers do not have satisfactory knowledge on newly introduced vaccines. This study also showed that the barriers of completion of child immunization especially in the rural area can be traceable to poor knowledge, attitude and perception of health facility support. This is congruent with the findings of Coreil et al, 1989 [40].

## Conclusion

Immunization coverage in the urban community was better than that of the rural community. The result of this study has clearly indicated that mothers in Nigeria have improved on taking their children for immunization in both rural and urban area. This suggests that immunization uptake in rural community has also improved compared to previous reports. The challenge however is that children of women without education, that are poor in the rural area and in the middle class in the urban area, were not fully immunized, thus affecting the immunization picture in Keffi LGA. Women empowerment intervention is thus recommended, for the poor women, as well as improved female literacy level as knowledgeable mothers utilize child health services better (including immunization) and this has been linked positively with child survival practices. Appropriate information and education strategies should be put in place to further improve awareness about immunization; this will ensure that mothers, especially the uneducated and poor, immunize their children since low coverage will always draw back the efforts of fighting vaccine preventable diseases. The immunization schedule should made frequent and more flexible, outreach centers should also be created to accommodate busy mothers as ''mother too busy' and 'place of vaccination too far' were also important reasons for incomplete immunization.

**Unanswered questions and future research**: This study was about mothers' characteristics and how it affected the immunization status of the children under one. Fathers' socio demographic factors were not considered which could be focus for future research in an African setting.

### What is known about this topic

- Adeleye O.A Showed that a child was likely to be fully vaccinated if he or she was born in the hospital, was of third birth order or beyond and mother knows the benefit of immunization;
- Findings by centre for disease control and prevention showed that low educational level and low socioeconomic status of mothers were linked to low vaccine uptake in the majority of reviewed studies;
- Angela Oyo-ita observed that failure of the child to be fully immunized was associated with mother's lack of information on the importance of immunization.

### What this study adds

- The age of the mother was significantly associated with immunization status of the child;
- There was no significant difference in immunization practice of mothers from urban and rural communities, though practice is a bit better among the urban women;
- Mothers' knowledge on newly introduce vaccines was poor both in the Urban and the rural communities studied.
